# Supplementary material for: Differences in Neuropathology between Nitroglycerin-Induced Mouse Models of Episodic and Chronic Migraine
Source: Int J Mol Sci. 2024 Mar 26;25(7):3706. doi: 10.3390/ijms25073706 (PMC11011425; doi:10.3390/ijms25073706)
Supplement: Supplementary file 1 [file ijms-25-03706-s001.zip › ijms-2897306-supplementary.pdf]

International Journal of
Molecular Sciences

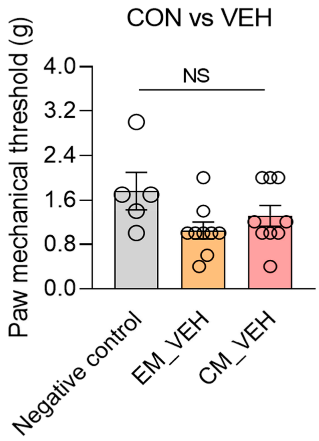

Supplementary Figure S1. Results represent the relative significance between the saline-treated control and vehicle groups. Statistical analysis was conducted using one-way ANOVA with post hoc Tukey test: ns, not significant.
